# Quiet Quitting Among Nurses: A Case Study in a Northern Italian Hospital

**DOI:** 10.3390/nursrep15070239

**Published:** 2025-06-29

**Authors:** Sara Rinaldi, Ester Pomarolli

**Affiliations:** 1 IRRCS Rizzoli Orthopaedic Institute, 40136 Bologna, Italy; 2 Hospital of Bolzano (SABES-ASDAA), 39100 Bolzano, Italy; ester.pomarolli@sabes.it

**Keywords:** quiet quitting, nurses, nursing, workplace, satisfaction

## Abstract

**Background/Objectives:** Quiet Quitting (QQ) describes a state of reduced workplace engagement characterized by a lack of motivation and initiative. Employees practicing QQ typically limit their efforts to fulfilling only their assigned tasks, expending the minimum necessary energy, while still ensuring the completion of their core responsibilities. QQ is an emerging phenomenon in healthcare settings following the COVID-19 pandemic and is still relatively unexplored in the literature. The aim of this study is to assess QQ among nurses in a northern Italian hospital. **Methods:** A cross-sectional study with a convenience sample of 91 nurses in a single-specialist orthopedic hospital was conducted. Data were collected in August 2024 using an online form of the Quiet Quitting Scale (QQS). Demographic data were collected, including gender, age, years of work experience and department. **Results:** A response rate of 83.50% was recorded. Data analysis revealed that 46.06% of the sample (35 nurses) was identified as quiet quitters. Fewer years of service and a younger age were associated with a higher probability of Quiet Quitting. The highest average score was observed for “Lack of motivation” subscale at 2.40 (SD = 1.14). **Conclusions:** The findings establish QQ as a significant issue requiring urgent attention in healthcare. To guarantee high-quality nursing care, healthcare organizations must prioritize interventions focused on enhancing employee support and work environment. Longitudinal research is necessary to explore the long-term effects of QQ on patient outcomes and to evaluate the success of targeted interventions.

## 1. Introduction

The aftermath of the COVID-19 pandemic has cast a long shadow over healthcare systems globally, exacerbating an already critical nursing shortage [1]. Among those remaining in clinical practice, there is a palpable sense of fatigue and job dissatisfaction, characterized by a dwindling passion for their work [2,3]. The intense and prolonged demands of high-acuity care during the COVID-19 pandemic resulted in widespread fatigue, psychological disengagement, and burnout among nurses [4]. Consequently, an increasing number of nurses are now performing only the strictly required tasks, minimizing their effort during working hours [5,6]. The phenomenon of quiet quitting (QQ) has become pervasive in hospitals, where employees, without consciously realizing it, are silently disengaging from their work [7]. Quiet quitters do not quit their jobs but limit their work to the bare minimum, ensuring they fulfill their duties without compromising their job security [8]. This behavior, characterized by a strict adherence to assigned tasks and working hours, differs distinctly from burnout or general job dissatisfaction, as it involves a deliberate decision to disengage while maintaining an outward appearance of compliance [5]. The contemporary reality for nurses in Italy, particularly within the hospital environment, presents a complex web of challenges: relentless workloads often coupled with inadequate compensation, persistent staff turnover involving less experienced colleagues, consistently stressful work rhythms, a precarious work–life balance, limited recognition and appreciation for their crucial role, and increasingly demanding interactions with patients and their families [9,10,11]. Faced with these pressures, nurses may feel compelled to stay in their current roles, deeming a job change too arduous. Consequently, they may decide to reduce their efforts on the ward by performing the minimum required to fulfill their duties. Quiet quitting, therefore, emerges as an alternative coping mechanism: a retreat from ambition where work is performed solely to avoid repercussions, prioritizing self-preservation and a semblance of work–life balance [2]. The main attitude of the quiet quitter is disengagement, encompassing a lack of interest and absence of passion for one’s work, resulting in a perceived irrelevance of expending extra effort [3]. While burnout and job satisfaction have been extensively explored in research, quiet quitting represents a relatively novel concept that has only recently begun to garner scholarly attention [5]. The existing literature on this significant issue remains sparse, with investigations primarily emerging in the post-pandemic era. Notably, the understanding of this phenomenon within the Italian hospital context and among Italian healthcare professionals remains particularly limited. This study aims to shed light on the prevalence and impact of quiet quitting among nurses in a Northern Italian Hospital, a profession that has been particularly hard-hit by the pandemic. Understanding the factors driving this disengagement is essential to inform the development of targeted interventions for improved employee well-being, job satisfaction, patient care quality, and a more supportive nursing environment [12].

To the best of our knowledge, this is the first study that has investigated the impact of quiet quitting in Italian hospitals, offering novel insights into this critical issue within the Italian healthcare landscape.

## 2. Materials and Methods

### 2.1. Study Design

A descriptive cross-sectional study was conducted on a convenience sample of 91 nurses working in various departments of a single-specialist orthopedic hospital of Northern Italy. An online form of the study questionnaire was created with Google Forms and then disseminated via institutional email. The data were collected in August 2024. The Quiet Quitting Scale (QQS) [13] was used to measure the level of quiet quitting. This scale consists of nine items (Table 1) that assess the three core components of the phenomenon: detachment (four items), lack of initiative (three items), and lack of motivation (two items). Items were scored on a five-point Likert scale from strongly disagree to strongly agree or from never to always. The QQS score is calculated by averaging the answers to the five items. Thus, the QQS score is a value between 1 and 5. We used the suggested cut-off point of 2.06 [8,14]: higher scores obtained at the Quiet Quitting Scale consider the subject a quiet quitter, and lower scores not a quiet quitter. For the Italian translation, a face validation was conducted through the hospital’s research center. The translation was accepted by the authors of the Quiet Quitting Scale and did not alter its content. Participants were asked to complete the Quiet Quitting Scale anonymously and provide demographic information, including gender, age, years of nursing experience, and current work unit.

### 2.2. Ethical Issues

Authorization to conduct the study has been granted to the management of the hospital institution and the faculty committee. When completing the questionnaire, the participants gave their informed consent to the processing of their data. The study was conducted on a voluntary and anonymous basis: the authors were not able to trace the identity of the participants. The study was conducted in accordance with the principles of the Declaration of Helsinki.

### 2.3. Statistical Analysis

Descriptive statistical methods were applied to analyze the data. The data analysis was performed using IBM SPSS Statistics version 25. Categorical variables are presented as frequencies and percentages. Continuous variables are reported using the mean, standard deviation (SD), minimum, and maximum values. The relationship between the variables was calculated using Pearson’s correlation coefficient and represented using scatter plots with a polynomial trendline.

## 3. Results

### 3.1. Demographic Characteristics

The sample included 76 nurses (response rate: 83.5%). The observed proportion of non-respondents within the study sample can likely be attributed to the constrained data collection timeframe, which overlapped with the summer holiday period. Participants had a mean age of 37.84 years (SD = 8.6) and an average of 12.9 years of nursing experience (SD = 8.9). Most nurses were females (75%). These demographics are representative of the hospital’s nursing staff. Participants were also asked to indicate the specific Operational Unit in which they were working at the time of completing the questionnaire. A total of 11 units were involved and categorized into three major care areas. The detailed socio-demographic characteristics of the nurses are shown in Table 2.

### 3.2. Descriptive Results for the QQS and Its Subscales

The mean score on the QQS was 2.07 (SD = 0.67), within a range from 1.0 to 4.33 and a median value of 2. In the sample, 46.6% (N = 35) of nurses had a QQS score above the cut-off point of 2.06 and were considered quiet quitters. On the other hand, 53.94% (N = 41) had a QQS score below the suggested cut-off point and were described as non-quiet quitters. QQS and its subscales are presented in Table 3. Participants showed a lower mean score of 1.93 (SD = 0.71) for “Detachment” and a slightly higher mean of 2.03 (SD = 0.86) for “Lack of initiative.” The highest average score was observed for “Lack of motivation” at 2.40 (SD = 1.14), indicating that this dimension of quiet quitting was the most pronounced within the study group.

### 3.3. Quiet Quitter Characteristics and Demographic Relationships

Quiet quitters (N = 35) were younger, with an average age of 35.4 years, compared to non-quiet quitters (N = 41) whose average age was 39.9 years (Table 4).

**Figure 1:**
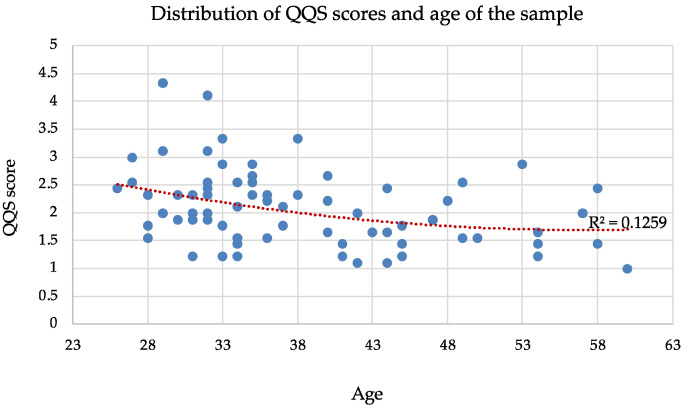
Distribution of QQS scores and age of the sample. Each blue circle represents a single individual in the sample, while the red dotted line indicates the overall trend between age and QQS score across the sample.

Therefore, the correlation between QQS score and these variables was investigated. The Pearson correlation coefficient between the scores of the Quiet Quitting Scale and the ages of the sample indicates a moderate negative correlation of −0.34. Although a very slight tendency for higher QQS scores in younger individuals might exist, it is not a strong or consistent pattern (Figure 1). The low R-squared value suggests that factors beyond age likely play a more significant role in determining an individual’s level of quiet quitting, warranting further investigation into these other potential influences.

**Figure 2:**
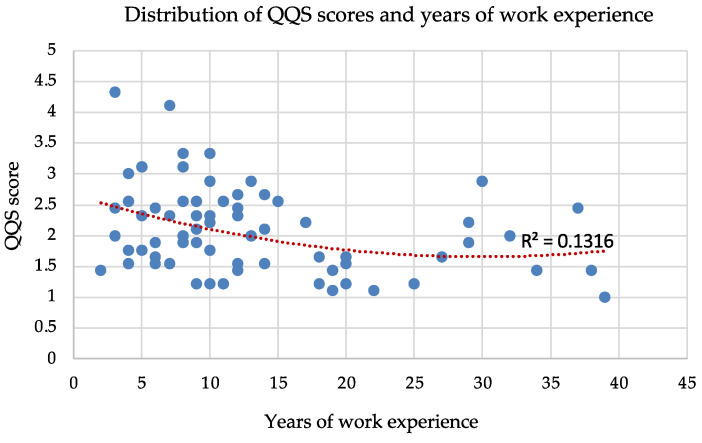
Distribution of QQS scores and years of work experience. Each blue circle represents a single individual in the sample, while the red dotted line indicates the overall trend between years of work experience and QQS score across the sample.

Figure 2 suggests a slight, non-linear relationship between years of work experience and QQS scores. QQS scores indicate a slight tendency to decrease as work experience increases up to around 10–15 years. This suggests that nurses with fewer years of service tend to report slightly higher QQS scores. However, the low R-squared value and the Pearson correlation of −0.32 indicate that years of work experience is not a strong predictor of QQS scores.

Median answer scores for every item are higher in the quiet quitters compared to the other group (Table 5). Quiet quitters are notably characterized by a greater tendency to delegate tasks, lower work motivation, and reduced inspiration. Furthermore, they are inclined to withhold work-related ideas due to a belief that these suggestions will not result in positive changes within the workplace.

## 4. Discussion

This study provides an initial insight into the prevalence and characteristics of quiet quitting among nurses in a Northern Italian hospital. The response rate of 83.5% suggests good engagement from the nursing staff, although the observed proportion of non-respondents may have been influenced by the data collection period coinciding with the summer holiday season, therefore potentially underrepresenting nurses who were on leave.

Our findings indicate that a substantial portion of the sample, 46.6%, met the criteria for being classified as quiet quitters, based on the adopted cut off point on the QQS [8,14]. This prevalence highlights the relevance of the phenomenon within the Italian healthcare context, although it is lower compared to findings in other studies [7,8,14,15], where the rate of quiet quitting among nurses reaches a maximum of 60% [16]. The pandemic has significantly affected this category of workers due to increased workload, staffing shortage, and emotionally demanding situations [17,18].

The findings of this research confirm that quiet quitters exhibit a pattern of reduced effort and disengagement, potentially driven by a sense of powerlessness and a desire to minimize their workload [14,16]. Emerging evidence suggests that the adoption of quiet quitting behaviors may reflect a conscious effort by individuals to prioritize well-being and manage workload, especially in the post-pandemic context where the importance of work–life balance has been amplified [12,17]. The item-level analysis further clarifies the distinguishing attributes of quiet quitters within our sample. Median response scores across all items were elevated for the quiet quitter group compared to their non-quiet quitting counterparts. Notably, the highest mean score observed for the “Lack of motivation” subscale suggests that this dimension is particularly pronounced among nurses, indicating a potential erosion of intrinsic drive towards their work. A recent study shows that organizational factors significantly impact these behaviors, with perceived unfair reward distribution being negatively linked to a lack of motivation [19].

The findings also revealed a moderate inverse relationship between age and QQS scores, indicating a propensity for younger nurses to report higher levels of quiet quitting. Similarly, a slight, non-linear trend indicated that nurses with fewer years of work experience tended to report higher QQS scores, particularly within the first 10–15 years of their career. However, the low R-squared values indicate that age and years of experience alone are not strong predictors of quiet quitting behavior. This, therefore, suggests that other unexamined factors likely play a more significant role in influencing a nurse’s level of disengagement. These findings are consistent with previous research [19], highlighting a greater propensity for quiet quitting among younger generations and those with shorter tenure, but remain a debated aspect within the existing body of research [20]. Several authors have noted a correlation between younger workers and a higher propensity to leave their jobs [20,21,22]. This inclination to seek new employment contrasts with the stance of the quiet quitter, who tends to remain in their current role rather than actively pursue alternative opportunities [6]. However, quiet quitting is often a temporary solution until a better job is found [7,23]. High turnover rates, often associated with younger and less experienced staff [22], are associated with poorer quality of care and increased patient mortality [23,24,25], making it a critical issue that should be actively mitigated. Furthermore, such turnover engenders instability within healthcare teams and fosters frustration among remaining personnel [25,26], thus representing a potential risk factor for the development of quiet quitting behaviors.

### 4.1. Implications for the Nursing Profession

Quiet quitting significantly endangers healthcare workforce stability and patient care, demanding immediate intervention. Addressing this requires robust measurement tools and identification of contributing factors like lack of organizational support and poor work environments [6,15,16,19,22,23]. Being a phenomenon that has become widespread in recent years (especially in hospital work environments) health management should be more aware and prepared to deal with the consequences of having quiet quitter employees and help nurses manage stress and heavy workloads [21]. The phenomenon should be assessed on a multifaceted level. Some authors suggest that healthcare organizations possess the capacity to facilitate the recovery of their personnel from work-related demands by promoting engagement in non-occupational activities post-work, such as mindfulness courses [27]. Psychological support and greater job recognition for those who are not satisfied and feel unsure of their work can help reduce the presence of quiet quitting [28]. Effective strategies for increasing job motivation involve improved leadership, collaborative environments, financial rewards, and recognition programs [29]. Healthcare leadership should prioritize the development of a positive working environment. Offering equitable and inclusive opportunities for personal and professional growth and advancement is key to preventing or limiting work disengagement [30,31,32]. To reduce disengagement and burnout, nurse managers and leaders should proactively engage in regular conversations with individual colleagues and teams, implementing workplace changes that foster a sense of value and shared purpose [31]. To cultivate a strong culture of learning and teamwork, organizations and managers should actively encourage all team members to participate in teamwork education programs [28,33]. Transformational leadership by nurse managers has been shown to reduce nurses’ turnover intentions [34], highlighting the impact of leadership styles on retention. Ultimately, improving working conditions and increasing awareness of quiet quitting are crucial for mitigating its detrimental effects [34,35].

Finally, ensuring quality care requires adequate staffing in terms of numbers and skills, alongside a sufficient and motivated workforce to guarantee safe assistance. Making the profession attractive through career progression and economic recognition of advanced skills is essential. At a national level, personnel policies are key indicators of NHS investment and are central to the operational functionality of healthcare facilities [32].

### 4.2. Limitations

Despite a strong response rate of 83.5%, the findings may be subject to selection bias and limited representativeness due to the small sample size (only 31.4% of hospital nurses) and the non-returned questionnaires. The limited data collection period, coinciding with the summer holiday season, likely contributed to the observed proportion of non-respondents in the study sample. It can be hypothesized that those who refused to complete the questionnaire likely had no interest in participating in the study, despite the very limited time required for completion and thus negligible energy expenditure. Therefore, in this case, the colleagues assumed a typical quiet quitter attitude, and their QQS score would likely have been positive, altering the results obtained from this study. It can be inferred that a selection bias likely occurred during the sample selection, as those who agreed to complete the questionnaire are colleagues willing to participate in an extra-work project and thus spend additional time beyond that required by their employment contract.

The reliability of the data may be compromised by the subjective nature of the responses, which are influenced by the timing of completion or personal contexts, such as vacations or life events [25]. To ensure more robust results, it would be appropriate to repeat the “Quiet Quitting Scale” at two-week intervals, as indicated by the literature [13].

The single-specialty orthopedic nature of the data collection hospital may limit our study. Nurses completing the QQS focus on a specific patient population, resulting in care and workload patterns that may differ significantly from other care settings.

This study offers a preliminary understanding of quiet quitting among nurses in a specific Italian hospital. Future research should explore the underlying factors contributing to this phenomenon in the Italian healthcare context, including workload, burnout levels, perceived organizational support, and opportunities for professional development. Longitudinal studies would be necessary to investigate the repercussions of quiet quitting on patient outcomes and professional turnover. Investigating potential interventions aimed at enhancing work engagement and addressing the specific concerns of younger and novice nurses may be crucial in fostering a more motivated and committed nursing workforce.

## 5. Conclusions

This study highlights the relevant prevalence of quiet quitting among nurses, which might undermine the efficiency and quality of healthcare services. Existing nursing working conditions and environments are seemingly conducive to this behavior. Health organizations must, therefore, acknowledge and assess this issue. While this study’s cross-sectional design and single-center focus limit generalizability, future research should investigate the factors contributing to quiet quitting, such as poor work environment, ineffective leadership, and inadequate personnel policies. Moreover, it is necessary to explore the impact of interventions for preventing the further widespread of QQ, such as resilience training, teamwork education programs, and reward enhancements.

## Figures and Tables

**Table 1:** The nine items included in the Quiet Quitting Scale.

Detachment
1. I do the basic or minimum amount of work without going above and beyond.
2. If a colleague can do some of my work, then I let him/her do it.
3. I take as many breaks as I can.
4. How often do you pretend to be working in order to avoid another task?
Lack of Initiative
5. I don’t express opinions and ideas about my work because I am afraid that the manager assigns me more tasks.
6. I don’t express opinions and ideas about my work because I think that work conditions are not going to change.
7. How often do you take initiative at your work?
Lack of Motivation
8. I find motives in my job.
9. I feel inspired when I work.

**Table 2:** Demographic characteristics of nurses (N = 76).

Characteristics	N	%
Gender		
Female	57	75
Male	19	25
Age ^a^	37.8	8.6
Years of work experience ^a^	12.9	8.9
Working department		
Oncology	20	26.3
Emergency	19	25
Other wards	37	48.7

^a^ Mean, standard deviation.

**Table 3:** Mean scores for QQs and its subscales.

	N	Mean	SD
Detachment	76	1.93	0.71
Lack of initiative	76	2.03	0.86
Lack of motivation	76	2.40	1.14
Total score	76	2.07	0.67

**Table 4:** Differences in age and years of work experience between quiet quitters and non-quiet quitters.

Variables	Quiet Quitter (N = 35)	Non-Quiet Quitter (N = 41)	Pearson Correlation Coefficient
Age, mean (SD)	35.4 (74)	39.9 (9.1)	−0.34
Years of work experience mean (SD)	10.9 (7.5)	14.7 (9.7)	−0.32

**Table 5:** Median answer scores quiet quitters vs. non quiet quitters.

Item	QQ Median Answer	Non QQ Median Answer	Range
Detachment			
I do the basic or minimum amount of work without going above and beyond.	2.1	1.2	1–5
2. If a colleague can do some of my work, then I let him/her do it.	3.0	2.0	1–5
3. I take as many breaks as I can.	2.6	1.9	1–5
4. How often do you pretend to be working in order to avoid another task?	1.7	1.2	1–5
Lack of Initiative			
5. I don’t express opinions and ideas about my work because I am afraid that the manager assigns me more tasks.	2.2	1.1	1–5
6. I don’t express opinions and ideas about my work because I think that work conditions are not going to change.	3.1	1.6	1–5
7. How often do you take initiative at your work?	2.6	1.8	1–5
Lack of Motivation			
8. I find motives in my job.	3.2	1.8	1–5
9. I feel inspired when I work.	3.3	1.7	1–5

## Data Availability

The data presented in this study are available upon request from the corresponding author.

## Public Involvement Statement

No public involvement in any aspect of this research.

## Guidelines and Standards Statement

This manuscript was drafted against the Strengthening the Reporting of Observational studies in Epidemiology (STROBE) guidelines for cross-sectional studies.
